# Circulating levels of mitochondrial oxidative stress-related peptides MOTS-c and Romo1 in stable COPD: A cross-sectional study

**DOI:** 10.3389/fmed.2023.1100211

**Published:** 2023-02-08

**Authors:** Carlos A. Amado, Paula Martín-Audera, Juan Agüero, Bernardo A. Lavín, Armando R. Guerra, Daymara Boucle, Diego Ferrer-Pargada, Ana Berja, Fernando Martín, Ciro Casanova, Mayte García-Unzueta

**Affiliations:** ^1^Department of Pulmonology, Hospital Universitario Marqués de Valdecilla, Santander, Spain; ^2^University of Cantabria, Santander, Spain; ^3^IDIVAL (Instituto de Investigación Biomédica de Cantabria), Santander, Spain; ^4^Department of Clinical Biochemistry, Hospital Universitario Marqués de Valdecilla, Santander, Spain; ^5^Unidad de Deshabituación Tabáquica (UDESTA), Servicio Cántabro de Salud, Santander, Spain; ^6^Servicio de Neumología-Unidad de Investigación, Hospital Universitario La Candelaria, Universidad de La Laguna, San Cristóbal de La Laguna, Spain

**Keywords:** COPD, MOTS-c, Romo1, exercise capacity, oxidative stress

## Abstract

**Background:**

MOTS-c and Romo1 are mitochondrial peptides that are modulated by oxidative stress. No previous studies have explored circulating levels of MOTS-c in patients with chronic obstructive pulmonary disease (COPD).

**Methods:**

We enrolled 142 patients with stable COPD and 47 smokers with normal lung function in an observational cross-sectional study. We assessed serum levels of both MOTS-c and Romo1 and associated these findings with clinical characteristics of COPD.

**Results:**

Compared with smokers with normal lung function, patients with COPD had lower levels of MOTS-c (*p* = 0.02) and higher levels of Romo1 (*p* = 0.01). A multivariate logistic regression analysis revealed that above-median MOTS-c levels were positively associated with Romo1 levels (OR 1.075, 95% CI 1.005–1.150, *p* = 0.036), but no association was found with other COPD characteristics. Below-median levels of circulating MOTS-c were associated with oxygen desaturation (OR 3.25 95% CI 1.456–8.522, *p* = 0.005) and walking <350 meters (OR 3.246 95% CI 1.229–8.577, *p* = 0.018) in six-minute walk test. Above-median levels of Romo1 were positively associated with current smoking (OR 2.756, 95% CI 1.133–6.704, *p* = 0.025) and negatively associated with baseline oxygen saturation (OR 0.776 95% CI 0.641–0.939, *p* = 0.009).

**Conclusions:**

Reduced levels of circulating MOTS-c and increased levels of Romo1 were detected in patients diagnosed with COPD. Low levels of MOTS-c were associated with oxygen desaturation and poorer exercise capacity using 6 min walk test. Romo1 was associated with current smoking and baseline oxygen saturation.

**Trial registration:**

www.clinicaltrials.gov; No.: NCT04449419; URL: www.clinicaltrials.gov. Date of registration: June 26, 2020.

## Introduction

Chronic Obstructive Pulmonary Disease (COPD) is one of the leading causes of mortality worldwide (1). Oxidative stress (OS) is one of the most important factors contributing to the pathogenesis and severity of COPD (2, 3). COPD is a heterogeneous disease with several potentially treatable traits that have been associated with disease prognosis (4) including muscle weakness (5), reduced exercise capacity (6), and increased oxygen desaturation (OD) during exercise (7). While several biomarkers of OS have been evaluated in COPD (8, 9), none are evaluated on a routine basis.

Mitochondrial open reading frame of the 12S ribosomal RNA type-c (MOTS-c) is a recently discovered micropeptide encoded by the mitochondrial genome. MOTS-c is produced primarily in mitochondria-rich tissues, including skeletal muscle (10). MOTS-c has also been detected in peripheral blood and has thus been tentatively identified as a circulating myomitokine (11). While circulating MOTS-c penetrates target cells rapidly, its mechanism of entry remains unknown (11). Receptors for the hormonal mitokine, humanin, have already been described; by contrast, no receptors have been identified that interact specifically with MOTS-c. *In vitro*, MOTS-c is detected primarily in the mitochondria; production is induced in response to glucose restriction (10) or OS (12). MOTS-c activates sarcoplasmic adenosine monophosphate-activated protein kinase (AMPK) and is then translocated to the nucleus, where it binds to antioxidant response element sequences in the promoter regions of nuclear factor erythroid 2-related factor 2 (NRF2) and other transcription factors, thereby modulating target gene activity (12). Results of recent research reveal that circulating levels of MOTS-c increase with acute intense exercise but decline in association with increasing age as well as obesity, coronary disease, diabetes mellitus, and kidney failure (13).

Reactive oxygen species modulator 1 (Romo1) is a redox-sensitive protein located in the inner mitochondrial membrane that regulates the integrity of mitochondrial cristae and mitochondrial shape under conditions of OS (14). Of note, defective cristae, abnormal branches, and swollen and fragmented organelles are frequently observed in respiratory epithelial cells isolated from COPD patients (3).

To the best of our knowledge, there are no previously published studies that document serum MOTS-c levels in patients diagnosed with COPD or smokers without COPD. On the other hand, the results of one small study documented higher serum Romo1 levels in COPD patients (*n* = 49) compared to healthy volunteers not matched by smoking status (*n* = 39) and a negative correlation between Romo1 levels and forced expiratory volume in the first second (FEV1) (%), but exercise capacity was not evaluated (15). Although MOTS-c and Romo1 are mitochondrial peptides, and are associated with chronic diseases, they have not been measured together before.

We hypothesized that, because of low exercise capacity and high OS characteristic of COPD, serum levels of both MOTS-c and Romo1 would be altered. We also hypothesized that circulating levels might be associated with specific outcomes of this disease, for example, exercise capacity.

## Methods

An observational cross-sectional study of patients receiving care at a COPD outpatient clinic in Spain was performed from November 2018 to December 2020. The study was reviewed and approved by the ethics committee of our institution (CEIm of Cantabria; 2018.276). Written informed consent was provided by all patients before entering the study. The study protocol was registered at www.clinicaltrials.gov (https://clinicaltrials.gov/ct2/show/NCT04449419).

### Participants

Participants were recruited at a dedicated COPD outpatient clinic during routine visits. Smokers without COPD (control group) were recruited from the smoking cessation clinics held at our institution.

The inclusion criteria were as follows: (i) patients diagnosed with COPD based on the Global Initiative for Chronic Obstructive Lung Disease (GOLD) guidelines (16) who were >40 years of age and (ii) sex, age, and smoking status- matched control patients who had not been diagnosed with COPD.

The exclusion criteria were as follows: (i) patients who experienced a COPD exacerbation within 8 weeks of inclusion in the study; (ii) patients undergoing pulmonary rehabilitation during or up to 6 months before inclusion in the study; (iii) patients previously diagnosed with coronary or peripheral artery disease or cancer; (iv) patients with respiratory diseases different from COPD; (v) patients with serum C-reactive protein levels > 2.5 mg/dL; and (vi) patients with a glomerular filtration rate < 50 ml/min/1.73 m^2^; (vii) patients using systemic corticosteroids within 8 weeks of inclusion in the study.

### Measurements

Body composition estimates were obtained using a bioelectrical impedance device (OMRON BF511, Omron, Japan). Spirometry and a six-minute walk test (6MWT) were performed according to the Spanish Society of Pneumology and Thoracic Surgery (SEPAR) protocol (17, 18). Maximum hand grip strength was measured with a GRIP-A hand dynamometer (Takei, Niigata, Japan). Disease-associated malnutrition was determined based on the European Society for Clinical Nutrition and Metabolism (ESPEN) consensus guidelines, including body mass index (BMI) <18.5 kg/m^2^ or 18.5–22 kg/m^2^, combined with a low fat-free mass index (FFMI) at <17 kg/m^2^ for men and <15 kg/m^2^ for women (19). Participants were categorized as having a high risk of exacerbation (HRE) at the time of entry into the study if they had two or more moderate or one severe COPD exacerbation during the previous year as per GOLD guidelines (17). Oxygen desaturation (OD) was defined as a fall in oxygen saturation (SpO_2_) ≥4% or an overall SpO_2_ <90% (20). Serum creatinine, albumin, uric acid, and creatine kinase levels were measured with IDMS-traceable enzymatic assays (Atellica^®^ Analyzer, Siemens, Germany).

Serum levels of MOTS-c were determined using sandwich immunoassay (Mitochondrial Open Reading Frame of the 12S rRNA-c: MOTS-c kit, CEX132Hu, Katy, Cloud-Clone Corp., TX, USA Lot number L210213153). Serum levels of Romo1 were determined using sandwich immunoassay (Reactive oxygen species modulator 1 (Human ROMO1) ELISA Kit, Elabscience^®^, TX, USA Lot number ET4ZB9UKKQ) as per the manufacturers' protocols.

Blood from each participant was collected early in the morning to avoid any confusion that might result from circadian changes in MOTS-c and ROMO-1 levels. Samples and patient data were preserved by Biobanco Valdecilla (PT17/0015/0019), integrated into the Spanish Biobank Network, and processed according to standard operating procedures with approval from both ethical and scientific committees.

### Statistical analysis

Normally distributed data are presented as means ± standard deviations (SDs). Non-parametric data are presented as medians with interquartile ranges. We calculated the sample size in *Stata Statistical Software: Release 15*. College Station, TX: StataCorp LLC) based on an α risk of 0.05 and a β risk of 0.2. Differences between groups were evaluated with unpaired *t*-tests (for parametric data) or Mann-Whitney tests (for non-parametric data). Normal distribution was determined using the Kolmogorov–Smirnov test. The creation of a dichotomized variable from MOTS-c and Romo1 levels with a cut-off at the median value resulted in the highest discriminative power for the outcomes under study as it resulted in the lowest Akaike information criterion value, similar to that observed in other studies (21, 22). We determined cross-sectional associations by univariate and multivariate logistic regression based on high (i.e., above the median) vs. low (i.e., below the median) values of baseline circulating MOTS-c and Romo1 with baseline characteristics of the patients. Using a similar model, we evaluated the primary outcomes of the study: baseline oxygen saturation, and two outcomes of the 6MWT [6 min walking distance (6MWD) and oxygen desaturation (OD)] using SPSS Software version 25.00 for PC. All *p*-values resulted from two-tailed tests with *p* < 0.05 as statistically significant.

## Results

### Characteristics of patients and controls

**Figure 1:**
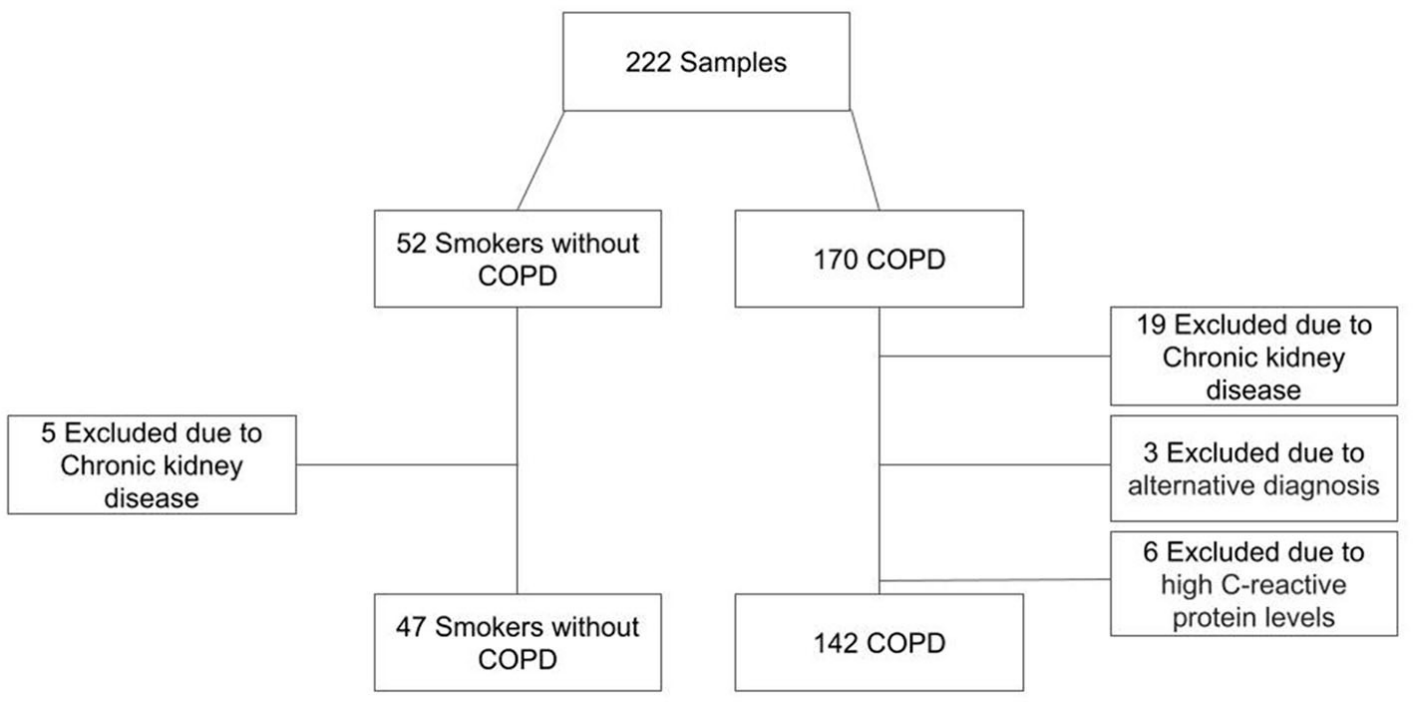
Flowchart for patient selection.

**Figure 2:**
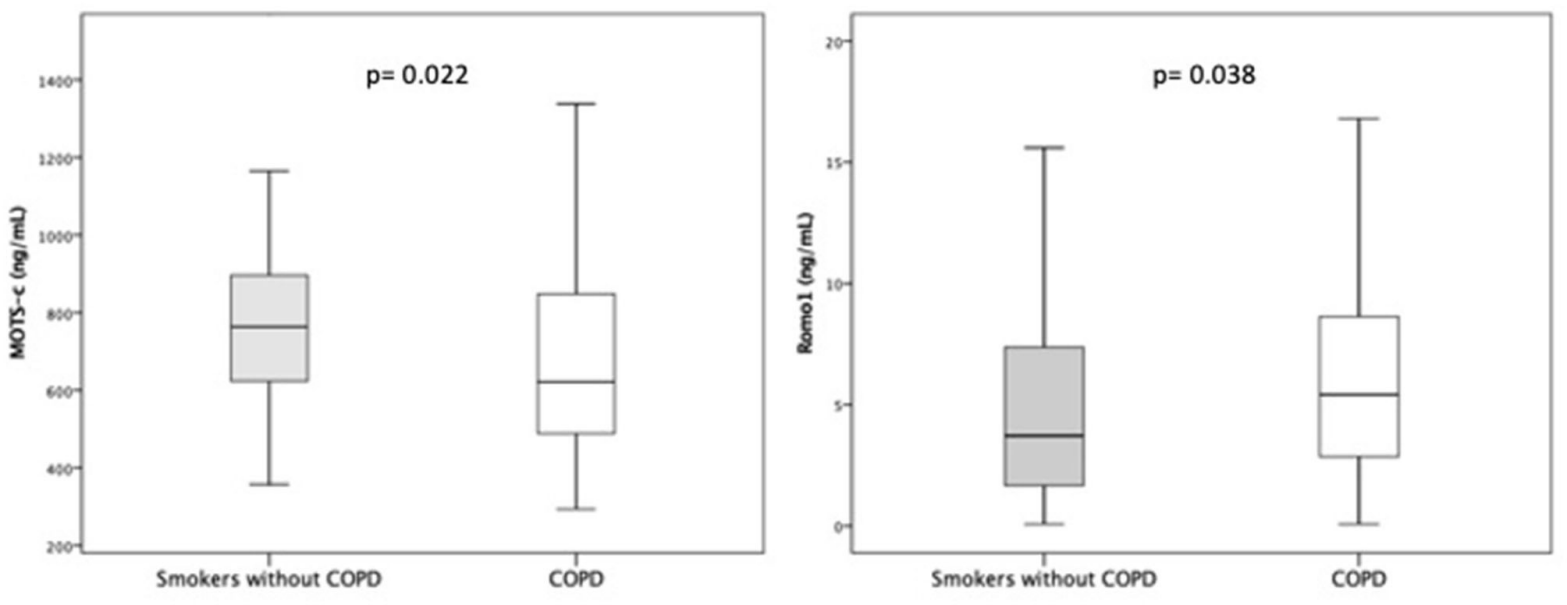
MOTS-c and Romo1 distribution in patients with COPD and smokers without COPD.

One hundred and forty-two COPD patients and forty-seven sex and age-matched controls (i.e., smokers who were not diagnosed with COPD) were enrolled in our study (Figure 1). Table 1 includes demographic, clinical, and biochemical data. The patient population was 66.2% men with a mean age of 67.5 ± 7.7 years. The COPD patient cohort included a substantial percentage of current smokers (30.6%); most had moderate to severe airway obstruction. The participants included in the control group had normal lung function, lower scores on COPD assessment tests (CATs), and were capable of longer six-minute walk distances (6MWD) than the COPD patients. As shown in Table 1 the percentage of current smokers was similar in COPD and control groups. The prevalence of type 2 diabetes mellitus (T2DM) which is a well-established cause of reductions in serum MOTS-c levels was similar in both groups (*p* = 0.842). Among our findings, MOTS-c levels were lower in the COPD group [median 622 ng/mL; interquartile range (IQR) 482–848 ng/mL] compared to controls (median 764 ng/mL; IQR 604–906 ng/mL; *p* = 0.022) (Figure 2). Our findings revealed no significant differences between serum levels of MOTS-c detected in patients with COPD either with (median 681 ng/mL, IQR 452–1150 ng/mL) or without T2DM (median 688 ng/mL, IQR 504–924 ng/mL; *p* = 0.853). Furthermore, serum levels of MOTS-c did not correlate with hemoglobin A1c (*r* = 0.165, *p* = 0.649) in patients with T2DM; thus, all COPD patients were evaluated as a single group.

**Table 1:** Demographic, clinical, and biochemical characteristics of control (smokers without COPD) and COPD patients.

**Variable**	**COPD *n =* 142**	**Smokers without COPD *n =* 47**	***p***
Age (years)	67.5 ± 7.7	65.6 ± 6.8	0.113
Sex Male *n* (%)	94 (66.2%)	31 (62.0%)	0.592
FVC (mL)	**2,723** **±814**	**3,429** **±861**	**<0.001**
FVC (%)	**83** **±20**	**102** **±18**	**<0.001**
FEV_1_ (mL)	**1,285 (900–1820)**	**2,635 (2072–3002)**	**<0.001**
FEV_1_ (%)	**52 (37–68)**	**98 (86–111)**	**<0.001**
FEV_1_/FVC	**49 (39–60)**	**75 (72–79)**	**<0.001**
Weight (Kg)	74.7 ± 16.2	74.3 ± 14.8	0.830
BMI (Kg/m2)	27 (24.1–31.6)	27.3 (25.1–29.8)	0.810
6MWD (m)	**445 (355–495)**	**525 (448–578)**	**<0.001**
Maximum hand grip strength (Kg)	31 (24–40)	33 (23–40)	0.667
FFMI (Kg/m^2^)	18.9 ± 2.7	19.2 ± 2.4	0.166
CAT score	**12 (7–18)**	**3 (1–5)**	**<0.001**
Charlson	1 (1–2)	1 (0–2)	0.092
mMRC score 0/I/II/III/IV	**39 (27.5)/43 (30.3)/38 (26.8)/22 (15.5)**	**38 (80.8)/8 (17.0)/1 (2.1)/0 (0) /0 (0)**	**<0.001**
Current smokers *n* (%)	43 (30.6)	22 (46.8)	0.059
Pack-years	41 (21–56)	40 (20–45)	0.098
Patients with malnutri-tion *n* (%)	**36 (25.4)**	**4 (8.5)**	**0.018**
GOLD 1/2/3/4 *n* (%)	19 (13.4)/59 (41.5)/47 (33.1)/17 (12.0)	–	–
GOLD A/B/C/D *n* (%)	49 (34.5)/39 (27.5)/13 (9.2)/41 (28.9)	–	–
High risk of exacerba–tion *n* (%)	56 (39.4)	–	–
ICS treatment *n* (%)	71 (50.0)	–	
Diabetes Mellitus *n* (%)	26 (18.3%)	8 (17.0%)	0.842
MOTS–c (ng/mL)	**622 (482–848)**	**764 (604–906)**	**0.022**
Romo−1 (ng/mL)	**5.42 (2.84–8.72)**	**3.72 (1.64–7.59)**	**0.038**
Albumin (g/dL)	4.80 ± 0.31	4.83 ± 0.28	0.681
Creatinine (mg/dL)	0.82 (0.70–0.96)	0.87 (0.71–0.98)	0.528
Uric acid (mg/dL)	6.19 ± 1.73	5.98 ± 1.38	0.464
CK (UI/L)	65 (45–95)	64 (41–113)	0.785

FVC, Forced Vital Capacity; FEV1, Forced expiratory Volume in the first second; mMRC, modified Medical Research Council Dyspnea score; CAT, COPD Assessment Test; ICS, Inhaled Corticosteroids; GOLD, Global initiative for Chronic Obstructive Lung Disease; BMI, Body Mass Index; FFMI, Fat Free Mass Index; 6MWD, 6- Minute Walking Distance; Bold font indicates statistical significance.

Serum levels of Romo1 were higher among those in the COPD group (median 5.42 ng/mL, IQR 2.84–8.72 ng/mL) vs. controls (median 3.72 ng/mL, IQR 1.64–7.59 ng/mL, *p* = 0.038) (Figure 2).

### Association of circulating levels of MOTS-c and Romo1 with baseline characteristics of COPD

Table 2 highlights the associations of MOTS-c and Romo1 with baseline characteristics of COPD and between both peptides. Multivariate logistic regression analysis showed that high levels of MOTS-c were positively associated with Romo1 levels (OR 1.075, 95% CI 1.005–1.150, *p* = 0.036), but no association was found with other COPD characteristics. On the other hand, multivariate logistic regression analysis confirmed that high levels of Romo1 were independently and positively associated with current smoking (OR 2.756, 95% CI 1.133–6.704, *p* = 0.025) and circulating MOTS-c levels (OR 1.001, 95% CI 1.000–1.003, *p* = 0.012) (Table 2).

**Table 2:** Univariate and multivariate logistic regression analysis for the associations between baseline chronic obstructive pulmonary disease characteristics and high levels of MOTS-c and Romo1.

		**High MOTS–c**				**High Romo**−**1**			
		**Univariate**		**Multivariate**		**Univariate**		**Multivariate**	
		**OR (95% CI)**	***p***	**OR (95% CI)**	***p***	**OR (95% CI)**	***p***	**OR (95% CI)**	***p***
Age (years)		0.994 (0.952–1.037)	0.777	0.979 (0.931–1.030)	0.412	0.980 (0.937–1.024)	0.361	0.988 (0.937–1.041)	0.648
**Sex**									
	Male	1		1		1		1	
	Female	1.660 (0.822–3.353)	0.157	0.436 (0.187–1.020)	0.065	0.880 (0.430–1.799)	0.726	1.553 (0.673–3.588)	0.302
**Smoking status**									
	Former-smoker	1		1		1		1	
	Current-smoker	1.222 (0.596–2.503)	0.584	1.093 (0.469–2.545)	0.837	**2.411 (1.119–5.195)**	**0.025**	**2.756 (1.133–6.704)**	**0.025**
**Exacerbation**									
	0	1		1		1		1	
	≥ 1	1.404 (0.725–2.719)	0.314	2.200 (0.932–5.193)	0.072	0.812 (0.408–1.617)	0.554	0.739 (0.315–1.734)	0.487
**Charlson**									
	1	1		1		1		1	
	2	0.743 (0.273–2.021)	0.561	0.992 (0.317–3.105)	0.989	0.726 (0.252–2.092)	0.554	0.767 (0.242–2.445)	0.656
	>2	1.479 (0.635–3.445)	0.364	2.331 (0.772–7.042)	0.133	0.444 (0.180–1.095)	0.078	0.328 (0.107–1.010)	0.052
FEV1 (%)		1.003 (0.987–1.019)	0.735	0.994 (0.963–1.025)	0.685	1.006 (0.989–1.023)	0.501	0.990 (0.960–1.020)	0.506
FVC (%)		1.007 (0.991–1.024)	0.400	1.017 (0.986–1.048)	0.282	1.008 (0.990–1.025)	0.387	1.022 (0.992–1.052)	0.158
Romo1 /MOTS-c (ng/mL)		1.050 (0.990–1.114)	0.104	**1.075 (1.005–1.150)**	**0.036**	**1.001 (1.000–1.002)**	**0.031**	**1.001 (1.000–1.003)**	**0.012**
Diabetes Mellitus		0.228 (0.353–1.943)	0.665	1.095 (0.377–3.184)	0.867	0.420 (0.167–1.057)	0.066	0.554 (0.184–1.671)	0.294

High MOTS-c levels ≥ 622 ng/mL; High Romo1 levels > 5.42 ng/mL; Exacerbation, Need for antibiotic or systemic corticosteroids; FEV1, Forced expiratory Volume in the first second; FVC, Forced Vital Capacity; Bold font indicates statistical significance.

### Low MOTS-c and high Romo1 levels as predictors of basal oxygen saturation, distance and oxygen desaturation in 6MWT

Forty-eight patients with COPD presented with Oxygen desaturation (OD). Among these, 33 exhibited low MOTS-c levels and 23 patients exhibited high Romo1 levels. Thirty-five patients walked <350 m during the 6MWT; this included 25 patients with low MOTS-c levels and 14 patients with high Romo1 levels. Multivariate logistic regression (Table 3) showed that low MOTS levels were significantly associated with OD (OR 3.25 95% CI 1.456–8.522, *p* = 0.005) and walking <350 meters (OR 3.246 95% CI 1.229–8.577, *p* = 0.018), but not with baseline O_2_ levels. On the other hand, high Romo 1 levels were negatively associated with baseline O_2_ levels (OR 0.776 95% CI 0.641–0.939, *p* = 0.009), but not with OD or walking <350 m (Table 4).

**Table 3:** Multivariate logistic regression analysis showing factors associated with baseline low MOTS-c.

	***B***	**Wald**	***p***	**OR**	**95% CI OR**	
					**Lower**	**Upper**
Baseline SatO_2_	−0.063	0.694	0.405	0.939	0.810	1.089
Oxygen Desaturation	**1.259**	**7.806**	**0.005**	**3.523**	**1.456**	**8.522**
6MWD < 350 meters	**1.178**	**5.643**	**0.018**	**3.246**	**1.229**	**8.577**

Low MOTS-c levels < 622 ng/mL; All variables adjusted by Age, Sex, Charlson Index, High risk of exacerbation (2 or more exacerbations during previous year or 1 previous admission), FEV1, Forced Expiratory Volume in the first second, smoking status. Oxygen desaturation was defined as ≥ 4% reduction between pretest and posttest arterial oxygen saturation (Δ SpO_2_ ≥ 4%) and posttest SpO_2_ < 90% measured by pulse oximetry. 6MWD, 6-Minute Walking Distance. Bold font indicates statistical significance.

**Table 4:** Logistic regression analysis showing factors associated with baseline high Romo1.

	***B***	**Wald**	***p***	**OR**	**95% CI OR**	
					**Lower**	**Upper**
Baseline SatO_2_	**−0.254**	**6.800**	**0.009**	**0.776**	**0.641**	**0.939**
Oxygen desaturation	0.064	0.018	0.892	1.066	0.425	2.673
6MWD < 350 meters	**–**0.311	0.353	0.552	0.733	0.263	2.043

High Romo1 levels >5.42 ng/mL; All variables adjusted by Age, Sex, Charlson Index, High risk of exacerbation (2 or more exacerbations during previous year or 1 previous admission), FEV1, Forced expiratory Volume in the first second, Smoking status. Oxygen desaturation (OD) was defined as ≥4% reduction between pretest and posttest arterial oxygen saturation (Δ SpO2 ≥ 4%) and posttest SpO2 < 90% measured by pulse oximetry. 6MWD, 6-Minute Walking Distance. Bold font indicates statistical significance.

## Discussion

The results of our study provide the first evidence that circulating levels of MOTS-c are lower in patients with COPD compared with otherwise healthy current smokers; by contrast, circulating levels of Romo1 were higher in patients with COPD. MOTS-c and Romo1 were associated with different COPD characteristics; low circulating MOTS-c levels were associated with worse 6MWD and oxygen desaturation; by contrast, high circulating levels of Romo1 were associated with active smoking and lower baseline levels of oxygen saturation. Interestingly we found a positive association between Romo1 and MOTS-c.

Circulating MOTS-c levels were reduced in COPD patients. Similar responses have been observed in other chronic diseases associated with OS, including T2DM (23), obesity with or without obstructive sleep apnea syndrome (24, 25), endothelial dysfunction/coronary artery disease (26), kidney failure (27), and multiple sclerosis (28). Interestingly, concomitant T2DM resulted in no further reductions in circulating MOTS-c levels compared to patients with COPD alone; these results suggest that the effects of these two diseases on MOTS-c levels are not additive. Low MOTS-c levels, as happens with “low T3 sick euthyroid syndrome”, may be a generalized non-specific response to many illnesses (29).

Reductions in circulating MOTS-c levels may result from mitochondrial damage. Alternatively, OS may induce profound sequestration of MOTS-c in the nucleus, thereby blocking its transfer into the peripheral circulation (12). Given that low levels of circulating MOTS-c have been reported in numerous diseases associated with OS, the second explanation may be the more likely of the two.

Lower levels of circulating MOTS-c were associated with shorter distances walked in the 6MWT, specifically with walking < 350 m, a parameter that is associated with the risk of death in COPD (6) and more profound OD. Exercise induces MOTS-c synthesis in healthy individuals (13, 30); MOTS-c in turn activates the synthesis of other enzymes (such as AMP-activated protein kinase) known to be induced by exercise (11, 31). On the other hand, it has been reported recently that MOTS-c promotes muscle differentiation of muscle progenitor cells (32). Thus, it is reasonable to hypothesize that individuals who are unable to induce MOTS-c synthesis will exhibit a comparatively low exercise capacity. One recent study evaluated the relationship between oxidative biomarkers associated with stable COPD and their role in exercise, identifying superoxide dismutase (SOD) as an independent determinant of performance in the 6MWT (33).

Our study shows that patients diagnosed with COPD have higher circulating levels of Romo1. This finding was also suggested previously in a cross-sectional study that included a small population of COPD patients (15). The results of our study reveal for the first time an important association between high circulating levels of Romo1 with current smoking and reduced oxygen saturation (i.e., factors intrinsically associated with oxidative stress), although no relationship with exercise capacity. Our data revealed a positive (albeit weak) association between high circulating levels of MOTS-c and Romo1. This relationship that has not been described previously, warrants further research and contrasts with the fact that low MOTS-c is associated with worse outcomes related with exercise capacity but high Romo1 is associated with low oxygen saturation, suggesting that each molecule relates to different characteristics of COPD. In a different model of respiratory disease, Ye et al. (34) have recently reported that serum Romo1 and ROS were increased in patients with obstructive sleep apnea syndrome.

Several of the strengths of this study are worth highlighting. First, this study was designed specifically to evaluate the impact of COPD on circulating levels of MOTS-c and Romo1 in a group of carefully selected and well-characterized patients without comorbidities (other than T2DM and asymptomatic coronary diseases) that might influence the results and a matched for current smoking control group of smokers without COPD (in order to avoid smoking as a potential confounding factor). Second, we considered a variety of factors and clinical characteristics of COPD that might have an impact on circulating levels of MOTS-c and Romo1.

Our study has several limitations. Most importantly, this type of study reveals associations but not causality. Any assessments of causality will require specifically designed *In vitro* and *in vivo* experimental studies. Furthermore, largely because of the complex pathophysiology of mitochondrial dysfunction and oxidative stress, a full understanding of this phenomenon will require consideration of a large collection of markers. Finally, our patient cohort was enrolled from a single center. Thus, these findings will need to be replicated in large multicenter trials with patients from a large range of socio-demographic settings and who exhibit a variety of comorbidities that might influence the results.

## Conclusion

Our study provides the first evidence of reductions in circulating levels of MOTS-c levels in patients diagnosed with COPD, lower MOTS-c is associated with lower 6MWD and higher rate of oxygen desaturation. Our study also revealed increases in levels of Romo1 in COPD patients that are associated with current smoking and baseline oxygen saturation but are not associated with 6MWD or oxygen desaturation in 6MWT. Further studies will be needed to confirm our findings which will open new perspectives in the multidimensional management of COPD.

## Data availability statement

The raw data supporting the conclusions of this article will be made available by the authors, without undue reservation.

## Ethics statement

The studies involving human participants were reviewed and approved by the Ceim of Cantabria. The patients/participants provided their written informed consent to participate in this study.

## Author contributions

Guarantor of the paper: CA. Conceptualization: CA and MG-U. Data curation: DF-P, CA, PM-A, and FM. Formal analysis: DF-P and CA. Project administration: CA and PM-A. Methodology: CA, MG-U, DB, AB, and AG. Resources: CA, MG-U, and AB. Visualization: CA and BL. Supervision: MG-U and PM-A. Software: DB. Writing—original draft: CA and CC. Writing—review and editing: CA, PM-A, AB, BL, AG, DB, and CC. All authors contributed to the article and approved the submitted version.
